# Bacterial Community Dynamics Distinguish Poultry Compost from Dairy Compost and Non-Amended Soils Planted with Spinach

**DOI:** 10.3390/microorganisms8101601

**Published:** 2020-10-18

**Authors:** Deborah A. Neher, Marie A. Limoges, Thomas R. Weicht, Manan Sharma, Patricia D. Millner, Catherine Donnelly

**Affiliations:** 1 Department of Plant and Soil Science, University of Vermont, Jeffords Hall, 63 Carrigan Drive, Burlington, VT 05405, USA; tweicht@uvm.edu; 2 Department of Nutrition and Food Sciences, University of Vermont, Marsh Life Science, 109 Carrigan Drive, Burlington, VT 05405, USA; marie.limoges@uvm.edu; 3 United States Department of Agriculture Research Service, 10300 Baltimore Ave, Beltsville, MD 20705, USA; manan.sharma@usda.gov (M.S.); pat.millner@usda.gov (P.D.M.)

**Keywords:** compost amendment, microbial community, ecoenzymes, poultry litter compost, dairy manure compost

## Abstract

The aim of this study was to determine whether and how poultry litter compost and dairy manure compost alter the microbial communities within field soils planted with spinach. In three successive years, separate experimental plots on two fields received randomly assigned compost treatments varying in animal origin: dairy manure (DMC), poultry litter (PLC), or neither (NoC). The composition and function of bacterial and fungal communities were characterized by the amplicon sequencing of marker genes and by the ecoenzyme activity, respectively. The temporal autocorrelation within and among years was adjusted by principal response curves (PRC) to analyze the effect of compost on community composition among treatments. Bacteria in the phylum Bacteriodetes, classes Flavobacteriia and Spingobacteriales (*Fluviicola*, *Flavobacteriia*, and *Pedobacter*), were two to four times more abundant in soils amended with PLC than DMC or NoC consistently among fields and years. Fungi in the phylum Ascomycota were relatively abundant, but their composition was field-specific and without treatment differences. The ecoenzyme data verify that the effects of PLC and DMC on soil communities are based on their microbial composition and not a response to the C source or nutrient content of the compost.

## 1. Introduction

In 2015, the use of manure-derived fertilizer occurred in 11.7% of U.S. fruit and vegetable farms, a significant 2.6% increase from six years prior. Much of this increase is attributable to the growing popularity of composted manure, which increased in use by 6.2% in the same period [1]. Farmers choose compost amendments based on price unless there is a convincing reason to avoid a material—e.g., it is known to contain harmful abiotic or biotic contaminants. Many farmers in the northeast of the U.S. choose poultry litter compost because it is the least expensive option and is on the list of approved materials for organic farmers [2]. Kyakuwaire et al. [3] present a compelling review that addresses the safety considerations of using non-composted poultry litter as an organic fertilizer. 

Compost is a controlled aerobic, microbially driven decomposition process [4]. The United States Department of Agriculture National Organic Program stipulates that windrow compost piles maintain temperatures between 55 and 77 °C for a minimum of 15 days and are turned a minimum of five times to ensure lethal conditions to inactivate microbial pathogens [5]. However, if compost is allowed to mature and cure, the carbon compound composition of the final product differentially supports a consortium of microorganisms which colonize the compost during the cooling phase of the process, some of which are antagonistic to phyto- and enteric pathogens [6,7]. The bacteria and fungi in the consortia have evolved defenses (against other microbes) that can be harnessed to target and suppress plant pathogens. Likewise, these saprophytic microbes in compost may also suppress foodborne pathogens through nutrient competition, antibiosis, inhibition, and predation [8]. Composts can serve as a means to introduce heterotrophic microbes that could alter indigenous soil microbiomes through antibiotic production, siderophores to sequester nutrients, or the production of enzymes that degrade cell walls [6]. 

There is an increasing trend to feed food waste to poultry as a means to divert edible material to a useful purpose prior to sending it to a landfill or composting [9]. Food waste may contain bacteria known as human pathogens that may harbor antibiotic resistance genes or other contaminants, including heavy metals [10]. Agricultural composts and soils represent a major contact point between the environment, animals, and humans. Fortunately, the composting of manure kills most pathogens of concern for food safety and crops (bacterial, fungal, oomycota, protozoan, nematode) [11,12]. Good agricultural practices suggest that non-composted manure should only be applied when the interval between the application and harvest is greater than four months. Most studies are performed under laboratory conditions with artificially inoculated samples. Such studies reveal taxonomic and functional diversity within soil microbial communities, yet little is known about the ecological function of these microbes [13,14], especially under field conditions. 

The aim of this study was to determine whether and how poultry litter and dairy manure composts alter microbial communities within field soils planted with spinach, and to identify which taxa explain any differences. We hypothesized that poultry litter compost will create conditions more favorable to the viability of copiotrophic organisms than dairy manure compost or unamended soils. For this study, we sequence marker genes of 16S and ITS to characterize the community composition of bacteria/archaea and fungi, respectively, and ecoenzymes to the relative availability of nutrients in compost-amended soils necessary to sustain the microbial community [15].

## 2. Materials and Methods 

### 2.1. Field Experimental Design

Two fields in South Burlington, Vermont (44°26′37.4″ N, 73°11′23.2″ W), with sandy loam soil were used for the field trials, with new plots established in different locations in each of three field seasons (May to November 2015, 2016, and 2017). Both fields were in hay production for 10 years prior to the study. The “Lilac” field contained a Hinesburg B fine sandy loam soil with a pH of 6.2 and an organic matter content of 2.8%. The “Wheelock” field contained an Adams B loamy sand soil with a pH of 6.2 and an organic matter content of 2.4% (Table 1). 

Three soil treatments were applied to soil plots in three different years: (1) poultry litter compost (PLC), (2) dairy manure compost (DMC), and (3) control without compost (NoC). The treatments were arranged in a completely randomized design (2015) or a randomized complete block design (2016, 2017) in two fields. Within each field, the treatments were replicated 3, 5, and 4 times in 2015, 2016, and 2017, respectively. The experimental plots (1 × 2 m) were separated by 1.5 m buffer strips and tilled to a depth of 30 cm using a rototiller (Troy Bilt, Cleveland, Ohio, U.S.) to prepare the soil. The order of plot establishment was first applying compost (Table 1), followed by tillage at a 10 cm depth using a rototiller, planting spinach seeds, and tamping down to prepare a seed bed. 

### 2.2. Compost Treatments

DMC and PLC were obtained from commercial or research sources that regularly provide products with similar recipes and maturities (Table 1) [16]. Application rates were based on preliminary experiments, industry recommendations, and product availability (Table 2) [16,17]. DMC applications added 282, 101, and 180 kg total nitrogen ha^−1^ and 33, 12, and 21 kg total phosphorus ha^−1^ in 2015, 2016, and 2017, respectively. The PLC applications added 414, 210, and 377 kg total nitrogen ha^−1^ and 281, 143, and 256 kg total phosphorus ha^−1^ in 2015, 2016, and 2017, respectively. Compost was manually spread uniformly across the surface of each plot. Both composts represent animal manure and their bedding without other additives such as food waste or yard waste. DMC and PLC were processed by aerobic static pile and windrow, respectively, until the thermophilic requirements were met, followed by six months of curing by windrow. 

### 2.3. Agronomic Practices

Approximately 390 Hybrid savoy-leafed spinach (*Spinacia oleracea* L.) Reflect F1 seeds were planted by hand-broadcasting across each plot. In addition to spinach plants, weeds grew on all the plots to emulate the effect of the plant rhizosphere on the soil microbial community dynamics. Although the abundance of weeds was similar among all plots, the species tended to vary between fields. Plots were irrigated as needed to meet crop demands during the duration of the study.

### 2.4. Soil Sampling

Soil samples were taken at frequent intervals after planting and less frequently thereafter (Table 3). Each sample was a composite of three 10 cm deep (2.5 cm dia) soil cores taken in a stratified random sampling pattern per plot. All the soil samples were sieved through a 2 mm mesh prior to subsampling for different analyses. Subsamples for ecoenzymes and amplicon sequencing were frozen at −80 °C and processed later. A separate subsample of soil was dried at 90 °C to compute the gravimetric moisture for the standardization of activity measurements as the g^−1^ of dry weight of soil (gdw).

### 2.5. Bacterial and Fungal Community Composition 

DNA was extracted from 0.5 g of each soil sample using the Qiagen PowerSoil DNA Isolation kit (Germantown, Maryland, U.S.) following the manufacturer’s instructions with the modifications described by Lauber et al. [18]. The extracted DNA was PCR-amplified using 515F/806R primers targeted for the V4 region of the 16S rRNA gene for bacteria and archaea and ITS-1F/ITS-2R primers to amplify the ITS-1 spacer gene of 18S rRNA for fungi following the protocols described previously [19]. Sequencing was conducted on an Illumina MiSeq (2 × 150 bp chemistry) at the University of Colorado’s Next Generation Sequencing Facility. Reads were merged, demultiplexed, and quality-filtered using the UPARSE pipeline [20]. Sequences were clustered into operational taxonomic units (OTUs) at the >97% sequence similarity level, with the taxonomic identity of each OTU determined using the RDP classifier with a threshold of 0.5 [21] trained against either the Greengenes database for bacterial and archaeal 16S rRNA gene sequences [22] or the UNITE database for fungal ITS sequences [23]. Detailed methods are available [16]. Raw amplicon sequence data are available in the public Figshare database (URL: https://figshare.com/account/home#/projects/72326 and DOI:10.6084/m9.figshare.11286113). 

### 2.6. Microbial Ecoenzymatic Activity

The microbial nutrient acquisition was determined by a microplate technique of four substrates labeled with methylumbelliferone (MUB) or methylcoumarin (MC) [24]. Substrates were chosen to target cellulose (BG-β-1,4-glucosidase), chitin (NAG-β-1,4-N-acetylglucosaminidase), leucine (LUC-L-leucine aminopeptidase), or phosphomonoesters (AP-phosphatase) (Table S1). Fluorescence was converted to the nmols of substrate used h^−1^ incubated gdw^−1^ to yield the enzyme activity in h^−1^ gdw^−1^. The ecoenzyme activity was used to determine whether the compost contained adequate energy (carbon), nitrogen, and phosphorus for the microbial community to sustain itself [15]. 

### 2.7. Statistical Analysis

16S rRNA and ITS amplicon sequences were rarefied to depths of 4000 and 10,000 reads per sample, respectively, prior to computing downstream analyses. Protist and plant sequences were eliminated, and the only OTUs present in at least 20% of the samples were analyzed. Temporal autocorrelation within and among years was adjusted by principal response curves (PRC) to analyze the effect of compost on the community composition among treatments. Time was expressed as the three years in linear sequence to provide a standardized scale and easy comparison among years. Statistical significance was computed by the Monte Carlo permutation of both first ordination axis and all axes together using CANOCO ver. 5.12 software [25]. Distinguishing 16S taxa were those that emerged after filtering the PCR results for both fields combined and each field separately (Table S2).

A three-way ANOVA was performed to analyze the effect of year, field, and compost on the nutrient availability, transformed as the vector length or angle of C:N as a function of C:P. Vector length represents carbon availability, calculating the square root of the sum of the squared values of *x* and *y*,

Length = SQRT (*x*^2^ + *y*^2^), (1)

where *y* is C:N and *x* is C:P [26]. Values > 1.0 suggest a carbon limitation. If carbon is not limited (values < 1.0), then N or P is limited, as determined by computing the angle as the arc-tangent of the line between the plot origin (0,0) and the data point (*x, y*) [26]:

angle (degrees) = DEGREES [arctangent (*x*, *y*)]. (2)

## 3. Results

### 3.1. Microbial Composition of Dairy and Poultry Based Composts

**Figure 1:**
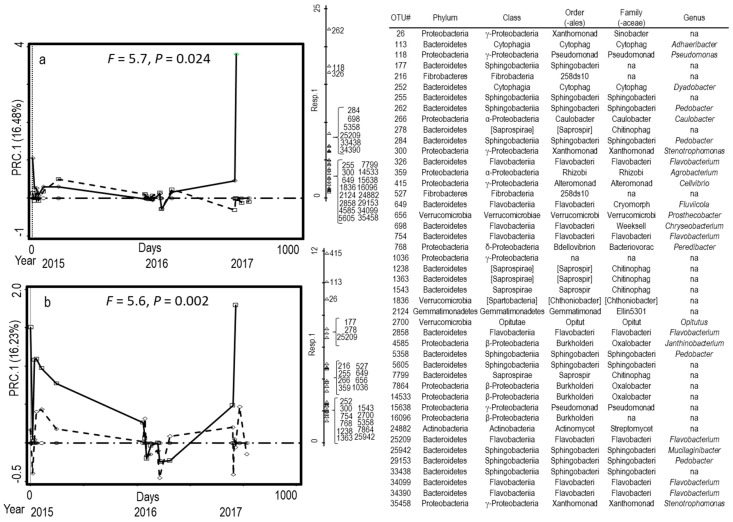
First principal response curve coefficient (PRC.1 with explained fitted variation in parentheses) of the 16S amplicon sequences for (**a**) Lilac and (**b**) Wheelock. Curves represent the deviation between a compost treatment (dashed: dairy manure; solid: poultry litter) from a non-amended control (dotted-dash) as a function of time, with 0 as the day of compost amendment in 2015 and the continuous calendar time until the end of the 2017 season. Symbols mark the sampling times. The weights of the 25 best-fit OTUs are shown on the right axis. Missing taxonomic information occurs if higher resolution was not available (na) for the OTU. Monte Carlo permutation tests permuting whole time series were applied to compute the statistical significance (*n* = 186 for Lilac, 181 for Wheelock).

**Figure 2:**
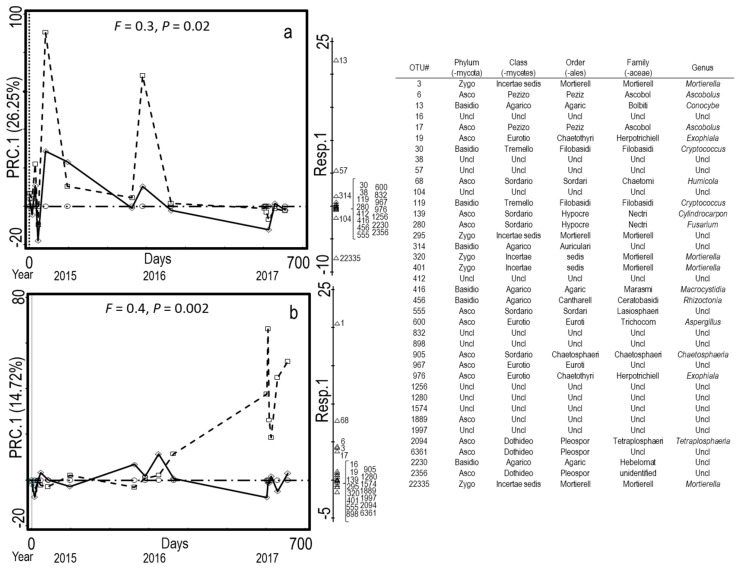
First principal response curve coefficient (PRC.1 with explained fitted variation in parentheses) of the ITS amplicon sequences for (**a**) Lilac and (**b**) Wheelock. Curves represent the deviation between a compost treatment (dashed: dairy manure; solid: poultry litter) from a non-amended control (dotted-dash) as a function of time, with 0 as the day of compost amendment in 2015 and the continuous calendar time until the end of 2017 season. Symbols mark the sampling times. The weights of the 25 best-fit OTUs are shown on the right axis. Missing taxonomic information occurs if a higher resolution was not available for the OTU (Unclassified: Uncl). Monte Carlo permutation tests permuting whole time series were applied to compute the statistical significance (*n* = 137 for Lilac, 165 for Wheelock).

Fluctuating seasonal patterns for each of three years emerged, although the amplitude of season varied among years. Compost treatment differences explained the variation in bacterial and fungal communities. First, PLC changed the bacterial communities in field soil more than DMC (Figure 1). The amplitude of the difference was greater in 2015 and 2017 than in 2016 (Figure 1). Compared to the NoC soil, the PLC amendments consistently increased Bacteriodetes orders Sphingobacteriales (*Pedobacter* spp.) and Flavobacteriia (*Flavobacterium* spp.) in both fields (Figure 1). Although the genera representing the γ-Proteobacteria varied by field, the PLC amendments accounted for a greater abundance compared to the DMC- or NoC-amended soils over time (Figure 1). Contrary to bacteria, the fungal communities in the DMC-amended soils contrasted those of the NoC-amended treatments more than with PLC (Figure 2). Basidiomycota *Conocybe* was prevalent in the Lilac field, whereas Ascomycota *Pseudaleuria* and *Humicola* distinguished the DMC-amended soils in the Wheelock field (Figure 2). Zygomycota *Mortierella* was associated with applications of PLC to the soils (Figure 2). 

**Figure 3:**
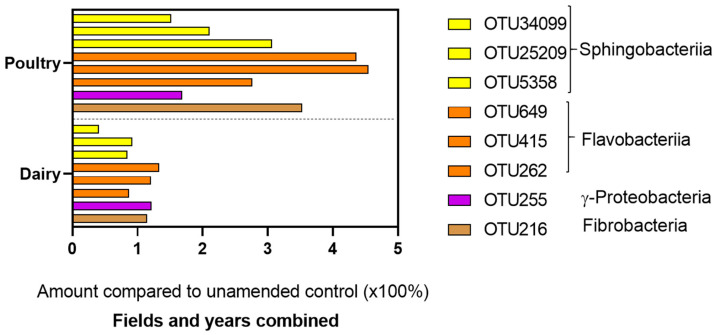
Order of magnitude in amplicon abundance compared to the no-compost control. Both fields and three years were combined. Bar colors represent phyla Sphingobacteriia (yellow), Flavobacteriia (orange), γ-Proteobacteria (purple), and Fibrobacteria (brown).

Bacterial communities of PLC-amended soils contrasted those of DMC- or NoC-amended soils across fields and years (Table 4, Figure 1). Soils amended with PLC were distinguished by an abundance of *Pedobacter* and unidentified genera in the Sphingobacteriaceae (Bacteroidetes) and *Flavobacterium* and *Fluviicola* in Flavobacteriaceae (Bacteroidetes). Order 258ds10 of Fibrobacteres and *Cellvibrio* of the γ-Proteobacteria were also characteristic of PLC treatments (Figure 1). Members of Sphingobacteriia, Flavobacteriia, γ-Proteobacteria, and Fibrobacteria amplicons were two to four times more abundant in soils amended with PLC than DMC or NoC (Figure 3). The fungal community composition of DMC- contrasted with the NoC-amended plots more than the PLC-amended plots, but there were no taxa that consistently distinguished the DMC- from PLC-amended soils across years and fields (Figure 2).

### 3.2. Microbial Nutrient Acquisition

**Figure 4:**
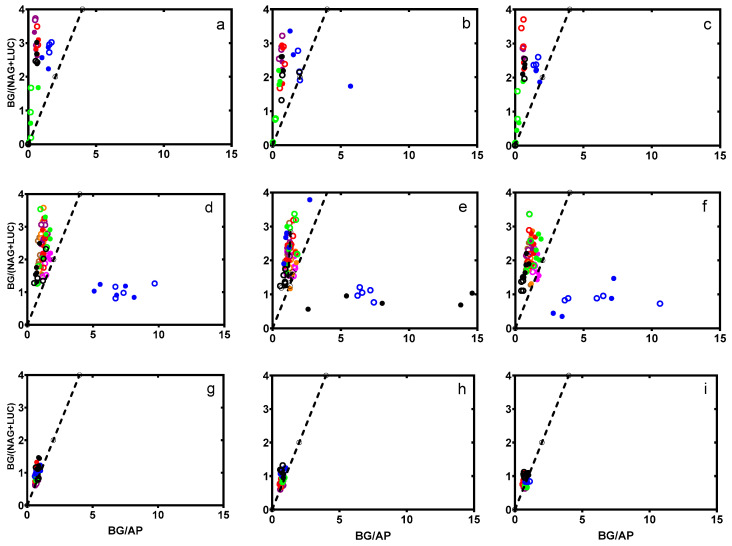
Ecoenzyme activity of C:N (*y*-axis) as a function of C:P (*x*-axis) over time, with a dashed line representing the 1:1 reference. Treatments are organized by column: (**a**,**d**,**g**) no compost, (**b**,**e**,**h**) dairy compost, and (**c**,**f**,**i**) poultry litter compost. C, N, and P activity were measured by BG: β-1,4-glucosidase, NAG: β-1,4-N-acetylglucosaminidase plus LUC: L-leucine aminopeptidase, and AP: phosphatase, respectively. Each row represents a study year: (**a**–**c**) 2015, (**d**–**f**) 2016, and (**g**–**i**) 2017. Within each panel, colors represent the sampling times within a year: (1) black; (2) blue; (3) green; (4) red; (5) purple; (6) orange; (7) fuchsia; (8) brown. Fields are labeled as Lilac soil (open circle) and Wheelock soil (closed circle).

Both the carbon availability and nutrient limitation differed by year (*p* < 0.001) but not by field or compost treatment (*p* > 0.2, Figure 4). The vector length values exceeded 1.0 (i.e., indicating carbon limitation). The angle values, which indicate nutrient availability, declined to less than 1.0, which indicates a shift from nitrogen to phosphorus limitation as the experiment progressed throughout the three-year study.

## 4. Discussion

This study demonstrates the differential effects of poultry litter-based and dairy manure-based composts on indigenous soil bacterial and fungal microbial communities in field soils across three different years and two fields with contrasting soil type. Evidence supporting the hypothesis that poultry litter compost creates conditions favorable to copiotrophic organisms is presented.

### 4.1. Microbial Composition of Dairy and Poultry Manure-Based Composts

Both Bacteriodetes and Proteobacteria are commonly found in soils and are associated with composts made with animal manure [7,27]. Bacteroidetes are common gut microbiota of both cows and birds [28,29]. In addition to Bacteroidetes, Firmicutes and γ-Proteobacteria are common in chicken ceca [28]. There is increasing evidence that Bacteriodetes plays a crucial role in producing polymer-degrading enzymes that degrade a diverse array of polysaccharides, proteins, and chitin related to the cell walls of plants and fungi [29,30]. The relative abundance of Bacteriodetes correlates positively with pH [18]. Gut species are mostly from the Bacteroidetes class, whereas the environmental species found in this study belong to the Flavobacteria, Cytophagia, and Sphingobacteria classes [30]. The variation in the polysaccharides that environmental Bacteriodetes can degrade is a measure of habitat breadth [30]. For example, there was a positive correlation between Bacteroidetes and β-1,4-glucosidase in this study, one of the ecoenzymes associated with the degradation of cellulose [29]. In contrast, Bacteroidetes orders Flavobacteriales and Sphingobacteriales degrade a variety of polysaccharides and proteins as C sources [31]. Soils amended with PLC contain greater abundances of Sphingobacteriales and Flavobacteriales than DMC or NoC soils. The positive correlation between PLC and Sphingobacteria in 2015 [16] was confirmed in the years 2016 and 2017. The presence of known denitrifiers and copiotrophs, such as Sphingobacteriales (e.g., *Pedobacter*), indicates a high N and C availability in PLC [32]. The increase in environmental Bacteroidetes is an indication that the conditions are more favorable to a copiotrophic community [16,33], which in turn is conducive to soil-borne fungal phytopathogens such as *Rhizoctonia solani* [17]. 

The ecoenzyme data of the study reported here verify that the effects of PLC and DMC on soil microbial communities are not simply a response to the C source or nutrient content of the compost. The genera *Pedobacter*, *Fluviicola*, *Flavobacterium*, and *Cellvibrio* are reputed to harbor antibiotic resistance genes [34]. Their multi-fold presence in PLC suggests that PLC could be a reservoir for resistance genes in agricultural environments. Specific feed blends for poultry are designed to promote the health and growth of birds. Before regulatory and market force changes were activated, these blends often contained arsenic [10,35] and/or veterinary antibiotic residues [36]. Heavy metals, antibiotics, and human pathogens pass intact through the digestive system to the fecal material [35,36], which represents a portion of poultry litter. Dairy cow manure can also harbor β-lactam-resistant bacteria even if the cows were not treated with antibiotics [37]. We have no direct evidence that either PLC or DMC contained antibiotic resistance genes (ARG) despite the presence of genera associated with ARG. In addition, the composting process is demonstrated to mitigate the persistence of ARG [10]. 

### 4.2. Microbial Nutrient Acquisition

Ecoenzymes are only secreted by microbes in soils when nutrients are scarce, because protein synthesis is an energy-intensive process [15]. Thus, the ecoenzyme activity in soil provides insight into the relative nutrient availability for the microbial community to sustain itself. In the current study, the only variability in ecoenzyme activity was found at the beginning of the season, likely a reflection of the disturbance caused by tilling [38]. Carbon availability and nutrient limitations suggest that soils in this study became increasingly C- and P-limited for soil microbes as the experiment progressed [15,16,26]. 

In the 2016 seasonal trial, the samples tended to be N-deficient initially and then shifted to be more P-deficient after 14 dpc (Figure 4). The magnitude of difference between the PLC and DMC amendments can be partially explained by the doses between years. Of the three years, 2016 plots received the smallest dose of both DMC and PLC. Specifically, PLC was applied at 50% of the amount in 2016 compared to 2015 and 2017. This was an artifact of the limited availability of the same source of poultry litter compost in 2016. The dose of DMC in 2015 was greater than in either 2016 or 2017. This shift to a P limitation response is also observed in maize, where the abundance of phosphate-mineralizing bacteria responds more to the dose (130 or 260 kg ha^−1^) than the type (organic or inorganic) of phosphorus fertilizer [36].

## 5. Conclusions

In comparison to unamended soil, PLC changed bacterial communities in field soil more than the DMC did. PLC consistently increased Bacteriodetes orders Sphingobacteriales and Flavobacteriia at both field sites in all three years. These bacteria exhibit copiotrophic lifestyles that respond to high concentrations of nitrogen. Plentiful nitrogen not only tips the nutrient balance to a phosphorus limitation but also favors pathogens that relate to food safety [39] and crop yields [40]. We propose that Bacteroidetes communities may be a useful risk assessment tool in systems with known pathogen pressures. Further research is necessary to compare the ARG loads associated with poultry versus dairy manure products and the impact they have on indigenous soil microbial communities. The differential impacts of manure source on compost products could affect farmers’ choice of compost amendments. 

## Figures and Tables

**Table 1:** Soil chemistry before the treatment applications and the properties of compost treatments.

Parameter *	Lilac Soil	Wheelock Soil	Dairy Manure Compost (DCM)	Poultry Litter Compost (PLC)
Total solids (%)	ND †	ND	62.7	81.5
Total carbon (%)	ND	ND	27.3	22.7
Total nitrogen (%)	ND	ND	1.87	3.09
Total potassium (%)	2.4 × 10^−3^	1.2 × 10^−2^	1.07	3.8
Total phosphorus (%)	2.8 × 10^−3^	1.4 × 10^−3^	0.22	2.1
Total calcium (%)	1.3 × 10^−1^	1.2 × 10^−1^	1.00	3.0
Total magnesium (%)	1.1 × 10^−2^	9.7 × 10^−3^	0.29	0.57
Total sodium (%)	5.3 × 10^−4^	6.0 × 10^−4^	0.2	1.4
Volatile solids (%)	ND	ND	54.0	50.2
pH	6.2	6.2	7.9	7.7
Bulk density (lbs yd^−3^)	ND	ND	430	910
Bulk density (kg m^−3^)	ND	ND	255.1	539.9
Conductivity (dS m^−1^)	ND	ND	7.1	29.1
C:N ratio	ND	ND	14.6	7.4
NH_4_-N (mg kg^−1^)	3.2	3.3	29.2	1510
NO_3_-N (mg kg^−1^)	15.8	19.5	1.11	816
Total boron (mg kg^−1^)	0.2	0.2	19.4	65.6
Total copper (mg kg^−1^)	0.2	0.2	237	458
Total iron (mg kg^−1^)	4.3	4.2	539	1390
Total manganese (mg kg^−1^)	4.8	5.5	51.3	634
Total zinc (mg kg^−1^)	2.7	1.9	57.8	540

*: Dry basis can be determined by dividing the values by the proportion of total solids. †: Not determined.

**Table 2:** Site conditions/rainfall is cumulative for the duration of the season.

Year	Season Duration (dpc) *	Cum Rainfall (cm)	Mean (SD) Air Temp (°C) †	Dairy Manure Compost (t ha^−1^)	Poultry Litter Compost (t ha^−1^)
2015	57	25.7	21.2 (2.86)	15.1	13.4
2016	64	15.8	21.3 (3.45)	5.4	6.8
2017	53	21.8	17.1 (5.11)	9.6	12.2

*: Days post compost. †: Mean + 1 standard deviation.

**Table 3:** Sampling schedule.

Year	Compost Addition	Ecoenzymes (dpc *)	DNA (dpc) †
2015	June 2	8, 16, 23, 30, 50, 65	16S and ITS: 8, 15, 23, 30, 49, 104 ‡
2016	May 31	2, 8, 15, 30, 36, 43, 57, 64	16S: 2, 8, 15, 30, 56, 63, 99 ‡ ITS: 2, 30, 99 ‡
2017	May 6	3, 6, 11, 27, 53	16S and ITS: 3, 6, 11, 27, 53

*: Days post compost additions. †: Dates chosen based on maximum and minimum phosphatase activity. ‡: Post season.

**Table 4:** Unique* 16S taxa of poultry litter compost identified as those common in both Lilac and Wheelock fields separately. *: See Tables S3 and S4 for a complete list of the most abundant classified 16S and ITS taxa identified in all treatments and both fields, respectively.

OTU	Phylum	Class	Order	Family	Genus
216	Fibrobacteres	Fibrobacteria	258ds10		
255	Bacteroidetes	Sphingobacteriia	Sphingobacteriales	Sphingobacteriaceae	
262	Bacteroidetes	Sphingobacteriia	Sphingobacteriales	Sphingobacteriaceae	*Pedobacter*
415	Proteobacteria	γ-proteobacteria	Alteromonadales	Alteromonadaceae	*Cellvibrio*
649	Bacteroidetes	Flavobacteriia	Flavobacteriales	Cryomorphaceae	*Fluviicola*
5358	Bacteroidetes	Sphingobacteriia	Sphingobacteriales	Sphingobacteriaceae	*Pedobacter*
25209	Bacteroidetes	Flavobacteriia	Flavobacteriales	Flavobacteriaceae	*Flavobacterium*
34099	Bacteroidetes	Flavobacteriia	Flavobacteriales	Flavobacteriaceae	*Flavobacterium*

## Supplementary Materials

The following are available online at https://www.mdpi.com/2076-2607/8/10/1601/s1: Table S1: Enzymes tested and associated soil substrates, experimental substrates, and positive controls [16], Table S2: Candidate taxa were those that emerged after filtering PCR results for both fields combined and each field separately for each the 16S amplicon genes. Taxa highlighted in yellow are those chosen for representation in Table 4; Table S3: Median percentage of sequences of the most abundant classified bacteria by compost treatment, Table S4: Median percentage of sequences of the most abundant classified fungi by compost treatment.
