# Supplementary material for: Bacterial Community Dynamics Distinguish Poultry Compost from Dairy Compost and Non-Amended Soils Planted with Spinach
Source: Microorganisms. 2020 Oct 18;8(10):1601. doi: 10.3390/microorganisms8101601 (PMC7603165; doi:10.3390/microorganisms8101601)
Supplement: Supplementary file 1 [file microorganisms-08-01601-s001.zip › Table S1.pdf]

Table S1. Enzymes tested and associated soil substrates, experimental substrates, and positive controls [16].

Enzyme	Organic Substrate (Target Nutrient)	Substrate Used	Positive Control
β-1,4-glucosidase (BG)	Cellulose (Carbon)	4-MUB- β-D-glucoside (Sigma #M2133)	4-methylumbilliferyl (Sigma #M1381)
Phosphatase (AP)	Phosphomonoesters (Phosphorous)	4-MUB-phosphate (Sigma #M8883)	4-methylumbilliferyl (Sigma #M1381)
β-1,4-N-acetylglucosaminidase (NAG)	Chitin (Carbon and Nitrogen)	4-MUB-N-acetyl- β-glucosaminide (Sigma #2133)	4-methylumbilliferyl (Sigma #M1381)
Leucine (LUC)	L-leucine aminopeptidase (Nitrogen)	L-leucine-7-amido-4-methylcoumarin (Sigma #L2145)	7-amido-4-methylcoumarin (Sigma #A9891)
