# Supplementary material for: Bacterial Community Dynamics Distinguish Poultry Compost from Dairy Compost and Non-Amended Soils Planted with Spinach
Source: Microorganisms. 2020 Oct 18;8(10):1601. doi: 10.3390/microorganisms8101601 (PMC7603165; doi:10.3390/microorganisms8101601)
Supplement: Supplementary file 1 [file microorganisms-08-01601-s001.zip › Table S2.pdf]

Table S2 Candidate 16S taxa that distinguish treatment differences identified through principal response curves (PRC). Results for both fields combined and each field separately are tallied by OTU number. Taxa highlighted in yellow are those chosen for representation in Table 4.

OTU number	Lilac alone top 25	Lilac alone top 50	Wheelock alone top 25	Wheelock alone top 50	Fields combined top 25	Fields combined top 50
33		x				
113			x	x		
118	x	x				
177			x	x	x	x
185					x	x
195		x				
216		x	x	x	x	x
227				x		x
252			x			x
255	x	x	x	x		x
255	x					
262	x	x	x	x	x	x
266			x	x	x	x
278			x	x		
284	x	x				
300	x					x
326	x	x		x		x
343						x
359			x	x		x
371				x		x
415		x	x	x	x	x
445					x	
445						x
471					x	
471						x
482						x
497						x
527			x	x	x	x
532						x
542						x
649	x	x	x	x	x	x
656			x	x	x	x

661						x
698	x	x				
701						x
751		x				
754						x
768			x			
1036			x	x		x
1154						x
1238						x
1363						x
1543						x
2083						x
2536						x
2700			x			x
2798						x
2858	x					
4585	x	x				
5358	x	x	x		x	x
6392						x
7799						x
7864			x			
10109						x
10751						x
14533	x	x				
15573				x		
15638	x	x				
19282						x
25209	x	x	x	x		
25388						x
25942						x
29153	x	x				
31579					x	x
33438	x	x				x
34099	x	x		x		x
34390	x	x				
35281						x
35458	x	x				x
