# Supplementary material for: Bacterial Community Dynamics Distinguish Poultry Compost from Dairy Compost and Non-Amended Soils Planted with Spinach
Source: Microorganisms. 2020 Oct 18;8(10):1601. doi: 10.3390/microorganisms8101601 (PMC7603165; doi:10.3390/microorganisms8101601)
Supplement: Supplementary file 1 [file microorganisms-08-01601-s001.zip › Table S3.pdf]

Table S3. Median percentage of sequences of the most abundant classified bacteria/archaea by compost treatment: without compost (NoC), dairy manure compost (DMC), poultry litter compost (PLC)

Kingdom	Phylum	Class	Order	Family	Genus	NoC	DMC	PLC
Bacteria	Acidobacteria	Acidobacteria-6	iii1-15			6.88	6.81	6.77
Bacteria	Verrucomicrobia	[Spartobacteria]	[Chthoniobacterales]	[Chthoniobacteraceae]	DA101	2.83	2.53	2.61
Bacteria	Acidobacteria	[Chloracidobacteria]	RB41			2.46	2.42	2.22
Bacteria	Bacteroidetes	[Saprospirae]	[Saprospirales]	Chitinophagaceae		1.68	1.71	1.56
Bacteria	Acidobacteria	[Chloracidobacteria]	RB41	Ellin6075		1.44	1.52	1.45
Bacteria	Proteobacteria	Gammaproteobacteria	Xanthomonadales	Sinobacteraceae		1.38	1.47	1.30
Bacteria	Gemmatimonadetes	Gemm-1				1.25	1.33	1.19
Archaea	Crenarchaeota	Thaumarchaeota	Nitrososphaerales	Nitrososphaeraceae	candidatus *Nitrososphaera*	1.29	1.26	0.93
Bacteria	Nitrospirae	Nitrospira	Nitrospirales	Nitrospiraceae	*Nitrospira*	1.16	1.13	1.01
Bacteria	Proteobacteria	Alphaproteobacteria	Sphingomonadales	Sphingomonadaceae	*Kaistobacter*	0.97	1.04	1.21
Bacteria	Proteobacteria	Deltaproteobacteria	Syntrophobacterales	Syntrophobacteraceae		0.86	0.86	0.80
Bacteria	Proteobacteria	Alphaproteobacteria	Rhizobiales	Hyphomicrobiaceae	*Rhodoplanes*	0.83	0.80	0.77
Bacteria	Proteobacteria	Gammaproteobacteria	Xanthomonadales	Sinobacteraceae	*Steroidobacter*	0.75	0.71	0.72
Bacteria	Proteobacteria	Betaproteobacteria	MND1			0.74	0.78	0.66
Bacteria	Proteobacteria	Betaproteobacteria	IS-44			0.52	0.53	0.51
Bacteria	Acidobacteria	Acidobacteriia	Acidobacteriales	Koribacteraceae		0.51	0.51	0.45
Bacteria	Proteobacteria	Alphaproteobacteria	Rhizobiales	Bradyrhizobiaceae	*Bradyrhizobium*	0.51	0.49	0.48
Bacteria	Gemmatimonadetes	Gemmatimonadetes	N1423WL			0.49	0.49	0.41
Bacteria	Nitrospirae	Nitrospira	Nitrospirales	0319-6A21		0.47	0.49	0.41
Bacteria	Proteobacteria	Alphaproteobacteria	Rhizobiales	Hyphomicrobiaceae	*Hyphomicrobium*	0.40	0.37	0.37
Bacteria	Proteobacteria	Gammaproteobacteria	Pseudomonadales	Pseudomonadaceae	*Pseudomonas*	0.38	0.32	0.43
Bacteria	Proteobacteria	Betaproteobacteria	Ellin6067			0.37	0.36	0.32
Bacteria	Proteobacteria	Betaproteobacteria	Burkholderiales	Oxalobacteraceae	*Janthinobacterium*	0.31	0.32	0.41
Bacteria	Proteobacteria	Gammaproteobacteria	Thiotrichales	Piscirickettsiaceae		0.27	0.32	0.30
Bacteria	Verrucomicrobia	[Pedosphaerae]	[Pedosphaerales]	auto67_4W		0.28	0.28	0.30
Bacteria	Proteobacteria	Alphaproteobacteria	Rhizobiales			0.29	0.28	0.27
Bacteria	Proteobacteria	Betaproteobacteria				0.25	0.30	0.28
Bacteria	Proteobacteria	Deltaproteobacteria	Myxococcales	Haliangiaceae		0.30	0.25	0.25
Bacteria	Proteobacteria	Alphaproteobacteria	Rhodospirillales	Rhodospirillaceae		0.27	0.27	0.26
Bacteria	Acidobacteria	S035				0.28	0.28	0.22
Bacteria	Proteobacteria	Alphaproteobacteria	Sphingomonadales	Sphingomonadaceae		0.26	0.25	0.26
Bacteria	Planctomycetes	Phycisphaerae	WD2101			0.25	0.23	0.24
Bacteria	Verrucomicrobia	Opitutae	Opitutales	Opitutaceae	*Opitutus*	0.20	0.23	0.23
Bacteria	Bacteroidetes	Cytophagia	Cytophagales	Cytophagaceae	*Adhaeribacter*	0.14	0.19	0.32
Bacteria	Bacteroidetes	Cytophagia	Cytophagales	Cytophagaceae		0.21	0.20	0.21
Bacteria	Proteobacteria	Betaproteobacteria	A21b	EB1003		0.21	0.21	0.18
Archaea	Crenarchaeota	Thaumarchaeota	Nitrososphaerales	Nitrososphaeraceae	candidatus *Nitrososphaera*	0.22	0.22	0.15
Bacteria	Proteobacteria	Deltaproteobacteria	Myxococcales			0.21	0.21	0.17
Bacteria	Proteobacteria	Alphaproteobacteria	Rhodobacterales	Hyphomonadaceae		0.19	0.18	0.21
Bacteria	Bacteroidetes	[Saprospirae]	[Saprospirales]	Chitinophagaceae	*Niabella*	0.18	0.20	0.18
Bacteria	Bacteroidetes	[Saprospirae]	[Saprospirales]	Chitinophagaceae	*Flavisolibacter*	0.16	0.19	0.19
Bacteria	Acidobacteria	Acidobacteria-6	iii1-15	mb2424		0.18	0.16	0.19
Bacteria	Acidobacteria	[Chloracidobacteria]	11-24			0.18	0.18	0.16
Bacteria	Proteobacteria	Betaproteobacteria	Burkholderiales	Comamonadaceae	*Methylibium*	0.18	0.18	0.16
Bacteria	Proteobacteria	Betaproteobacteria	SC-I-84			0.16	0.18	0.16
Bacteria	Chloroflexi	Ellin6529				0.18	0.17	0.14
Bacteria	Firmicutes	Bacilli	Bacillales			0.18	0.15	0.16
