# Supplementary material for: Bacterial Community Dynamics Distinguish Poultry Compost from Dairy Compost and Non-Amended Soils Planted with Spinach
Source: Microorganisms. 2020 Oct 18;8(10):1601. doi: 10.3390/microorganisms8101601 (PMC7603165; doi:10.3390/microorganisms8101601)
Supplement: Supplementary file 1 [file microorganisms-08-01601-s001.zip › Table S4.pdf]

Table S4. Median percentage of sequences of the most abundant classified fungi by compost treatment: without compost (NoC), dairy manure compost (DMC), poultry litter compost (PLC)

Phylum	Class	Order	Family	Genus	Species	NoC	DMC	PLC
Zygomycota	Incertae sedis	Mortierellales	Mortierellaceae	*Mortierella*	*elongata*	3.95	3.28	9.56
Zygomycota	Incertae sedis	Mortierellales	Mortierellaceae	*Mortierella*	*rishikesha*	5.45	3.40	4.92
Zygomycota	Incertae sedis	Mortierellales	Mortierellaceae	*Mortierella*		4.42	4.34	4.79
Ascomycota	Pezizomycetes	Pezizales	Ascobolaceae	*Ascobolus*		3.49	3.10	6.03
Zygomycota	Incertae sedis	Mortierellales	Mortierellaceae	*Mortierella*	*epicladia*	4.08	4.00	3.74
Ascomycota	Pezizomycetes	Pezizales	Pyronemataceae	*Pseudaleuria*		1.76	1.36	8.65
Ascomycota	Sordariomycetes	Microascales	Microascaceae	*Pseudallescheria*	*fimeti*	3.29	3.22	2.29
Zygomycota	Incertae sedis	Mortierellales	Mortierellaceae	*Mortierella*	*exigua*	2.92	2.68	3.14
Ascomycota	Sordariomycetes	Hypocreales	Nectriaceae	*Fusarium*		2.20	2.39	1.75
Zygomycota	Incertae sedis	Mortierellales	Mortierellaceae	*Mortierella*	*amoeboidea*	1.35	1.45	1.03
Ascomycota	Dothideomycetes	Pleosporales				1.44	1.32	1.03
Ascomycota	Pezizomycetes	Pezizales	Pyronemataceae			0.88	1.15	1.73
Basidiomycota	Agaricomycetes	Agaricales	Bolbitiaceae	*Conocybe*	*apala*	0.72	2.22	0.47
Ascomycota	Sordariomycetes	Hypocreales	Nectriaceae			1.17	1.18	0.87
Basidiomycota	Agaricomycetes	Cantharellales	Ceratobasidiaceae	*Ceratobasidium*		0.88	1.51	0.80
Ascomycota	Sordariomycetes	Sordariales	Chaetomiaceae	*Humicola*	*grisea* var. *grisea*	0.58	0.55	1.96
Zygomycota	Incertae sedis	Mortierellales	Mortierellaceae	*Mortierella*		0.78	1.10	1.05
Ascomycota	Sordariomycetes	Sordariales	Lasiosphaeriaceae			0.57	0.72	1.25
Zygomycota	Incertae sedis	Mortierellales	Mortierellaceae			0.39	0.46	1.57
Ascomycota	Dothideomycetes	Incertae sedis	Pseudeurotiaceae	*Pseudogymnoascus*	*roseus*	0.98	0.65	0.51
Ascomycota	Eurotiomycetes	Chaetothyriales	Herpotrichiellaceae	*Exophiala*	*equi*	0.93	0.73	0.48
Ascomycota	Dothideomycetes	Pleosporales	Pleosporaceae			0.72	0.87	0.49
Ascomycota	Eurotiomycetes	Chaetothyriales	Chaetothyriaceae	*Cyphellophora*	*vermispora*	0.62	0.96	0.45
Ascomycota	Eurotiomycetes	Onygeles				0.78	0.74	0.50
Ascomycota	Dothideomycetes	Pleosporales			DWS12m2_2	0.84	0.70	0.43
Ascomycota	Pezizomycetes	Pezizales	Ascobolaceae			0.19	0.32	1.40
Basidiomycota	Agaricomycetes	Agaricales	Marasmiaceae	*Marasmius*	*oreades*	0.48	1.01	0.39
Basidiomycota	Agaricomycetes	Cantharellales	Ceratobasidiaceae			1.20	0.29	0.40
Ascomycota	Dothideomycetes	Pleosporales	Pleosporaceae	*Alterria*		0.61	0.59	0.51
Basidiomycota	Agaricomycetes	Corticiales	Corticiaceae	*Waitea*	*Rhizoctonia zeae*	0.46	0.50	0.59
Basidiomycota	Tremellomycetes	Filobasidiales	Filobasidiaceae	*Cryptococcus*	*terreus*	0.58	0.47	0.47
Basidiomycota	Agaricomycetes	Agaricales	Marasmiaceae	*Marasmius*		0.83	0.45	0.22
Basidiomycota	Agaricomycetes	Cantharellales	Ceratobasidiaceae			0.72	0.35	0.40
Ascomycota	Dothideomycetes	Pleosporales	Phaeosphaeriaceae	*Phaeosphaeria*		0.39	0.75	0.31
Zygomycota	Incertae sedis	Mortierellales	Mortierellaceae	*Mortierella*	*antarctica*	0.53	0.66	0.25
Ascomycota	Sordariomycetes	Sordariales	Chaetomiaceae	*Chaetomium*		0.54	0.45	0.32
Basidiomycota	Agaricomycetes	Cantharellales	Ceratobasidiaceae	*Ceratobasidium*		0.47	0.43	0.35
Ascomycota	Dothideomycetes	Pleosporales	Incertae sedis	*Paraphoma*		0.40	0.57	0.28
Ascomycota	Sordariomycetes	Sordariales	Lasiosphaeriaceae			0.20	0.18	0.80
Ascomycota	Leotiomycetes	Helotiales	Hyaloscyphaceae	*Cistella*	KUS_F52527	0.41	0.50	0.24
Basidiomycota	Agaricomycetes	Agaricales	Strophariaceae	*Agrocybe*	*pediades*	0.31	0.67	0.17
Ascomycota	Sordariomycetes	Sordariales	Lasiosphaeriaceae	*Podospora*		0.43	0.36	0.33
Zygomycota	Incertae sedis	Mortierellales	Mortierellaceae	*Mortierella*	*polygonia*	0.40	0.37	0.34
Basidiomycota	Agaricomycetes	Agaricales	Agaricaceae	*Cyathus*	*stercoreus*	0.26	0.61	0.13
Ascomycota					sp 2 UFMGCB 5757	0.55	0.30	0.13
Ascomycota	Sordariomycetes	Sordariales	Chaetomiaceae			0.22	0.21	0.53
Basidiomycota	Agaricomycetes	Cantharellales	Hydceae	*Sistotrema*		0.33	0.35	0.27
Ascomycota	Dothideomycetes					0.34	0.38	0.23
Basidiomycota	Agaricomycetes	Agaricales	Psathyrellaceae	*Coprinellus*	*bisporus*	0.01	0.92	0.00
Ascomycota	Dothideomycetes	Pleosporales	Didymosphaeriaceae	*Roussoella*	*neopustulans*	0.30	0.34	0.26

Basidiomycota	Agaricomycetes	Auriculariales				0.32	0.46	0.12
Ascomycota	Orbiliomycetes	Orbiliales	Orbiliaceae	*Arthrobotrys*	*oligospora*	0.43	0.28	0.16
Ascomycota	Dothideomycetes	Pleosporales	Leptosphaeriaceae	*Leptosphaeria*	sp MH_2001	0.20	0.20	0.45
Ascomycota	Leotiomycetes	Helotiales				0.45	0.30	0.09
Basidiomycota	Agaricomycetes	Agaricales	Entolomataceae	*Entoloma*	*perumbilicatum*	0.43	0.28	0.13
Ascomycota					sp FL_2010c	0.33	0.27	0.23
Ascomycota	Dothideomycetes	Pleosporales	Phaeosphaeriaceae	*Phaeosphaeria*	sp TMS_2011	0.35	0.32	0.12
Ascomycota					sp r433	0.28	0.25	0.24
Zygomycota	Incertae sedis	Incertae sedis	Incertae sedis	*Modicella*	*reniformis*	0.19	0.39	0.19
Ascomycota	Sordariomycetes	Sordariales	Lasiosphaeriaceae	*Podospora*	sp 84_110A	0.24	0.34	0.18
